# Effects on the photon beam from an electromagnetic array used for patient localization and tumor tracking

**DOI:** 10.1120/jacmp.v14i3.4138

**Published:** 2013-05-06

**Authors:** Wei Zou, Ricardo Betancourt, Lingshu Yin, James Metz, Stephen Avery, Alireza Kassaee

**Affiliations:** ^1^ Department of Radiation Oncology University of Pennsylvania Philadelphia PA USA

**Keywords:** Calypso, attenuation, spoiling effect, IMRT, VMAT

## Abstract

One of the main components in a Calypso 4D localization and tracking system is an electromagnetic array placed above patients that is used for target monitoring during radiation treatment. The beam attenuation and beam spoiling properties of the Calypso electromagnetic array at various beam angles were investigated. Measurements were performed on a Varian Clinac iX linear accelerator with 6 MV and 15 MV photon beams. The narrow beam attenuation properties were measured under a field size of 1 cm×1 cm, with a photon diode placed in a cylindrical graphite buildup cap. The broad beam attenuation properties were measured under a field size of 10 cm×10 cm, with a 0.6 cc cylindrical Farmer chamber placed in a polystyrene buildup cap. Beam spoiling properties of the array were studied by measuring depth‐dose change from the array under a field size of 10 cm×10 cm cm in a water‐equivalent plastic phantom with an embedded Markus parallel plate chamber. Change in depth doses were measured with the array placed at distances of 2, 5, and 10 cm from the phantom surface. Narrow beam attenuation and broad beam attenuation from the array were found to be less than 2%–3% for both 6 MV and 15 MV beams at angles less than 40°, and were more pronounced at more oblique angles. Spoiling effects are appreciable at beam buildup region, but are insignificant at depths beyond $d_{\text{max}}$. Dose measurements in a QA phantom using patient IMRT and VMAT treatment plans were shown to have less than 2.5% dose difference with the Calypso array. The results indicate that the dose difference due to the placement of Calypso array is clinically insignificant.

PACS number: 87.56.‐v

## I. INTRODUCTION

With the development of radiotherapy delivery techniques such as intensity‐modulated radiation therapy (IMRT), image‐guided radiation therapy (IGRT), and volumetric‐modulated arc therapy (VMAT), internal organ localization accuracy becomes a great safety concern. Change in patient anatomy and motion from respiration, cardiac motion, and bowel mobility can cause interfraction and intrafraction tumor target movements and result in dosimetric consequences.^1^, ^2^, ^3^, ^4^, ^5^ A wireless electromagnetic Calypso system (Varian Medical Systems, Palo Alto, CA) was developed and clinically implemented for tumor localization and real‐time tracking during radiation treatment.^6^, ^7^, ^8^ It tracks the targets with implanted electromagnetic beacons in real time without generating ionizing radiation.^(6)^ Before and during each treatment session, an electromagnetic source and receiver coil array was placed above patient to determine the 3D positions of the beacons. The combination of the array position in the room and the beacon positions relative to the array provides continuous monitoring of the target position during treatment. Studies have demonstrated the robustness and accuracy of the Calypso system in localization and monitoring of the electromagnetic beacons in static and dynamic phantoms.^6^, ^9^, ^10^ Patient radiation target localization with Calypso system was demonstrated to agree well with a linear accelerator on‐board imaging system.^7^, ^8^, ^9^, ^11^ The Calypso system has been used to study the inter‐ and intrafraction tumor motion in various treatment sites including prostate,^12^, ^13^ pancreas,^(14)^ and lung.^(15)^ The motion data assessed from Calypso system were used for motion management and margin reduction to improve the target coverage and organ sparing in prostate radiotherapy.^16^, ^17^, ^18^, ^19^ A system that integrates the Calypso positioning information with a dynamic multileaf collimator tracking was also developed to reduce the interplay effect.^(20)^

**Figure 1:**
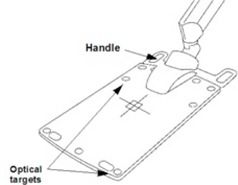
The electromagnetic array panel used in Calypso system (adopted from Varian Medical Systems, Palo Alto, CA, with permission).

The purpose of this article is to assess the dose effects introduced by the Calypso array that is placed above the patient through the treatment delivery. Figure 1 shows a drawing of this rectangular‐shaped array panel, which measured 63.5 cm in length, 44 cm in width, and 2.5 cm in depth. The major components of the array are embedded source coils that generate electromagnetic wave, and sensors that receive the signals transmitted from the beacons. The array also includes mechanical components such as handles, grips, and nine optical targets that communicate continuously with the in‐room optical system. Santanam et al.^(21)^ have studied effects of the array in their commissioning and quality assurance for clinical implementation of the Calypso system. They measured the transmission of the Calypso array at the normal incident beam angle and one oblique beam angle for both 6 and 18 MV energies, with an ion chamber embedded at the center of a 15 cm thick water‐equivalent plastic phantom. The transmission rate were found to be larger than 0.97 at both energies. The current article studies the array attenuation effects at various beam angles in narrow‐ and broad‐beam geometries. The methods used are similar to those used in couch attenuation studies.^22^, ^23^, ^24^ The beam spoiling effect of the array was addressed. The measurements with patient IMRT and VMAT plans were also performed to assess the dose differences in typical treatment deliveries with the Calypso system.

## II. MATERIALS AND METHODS

**Figure 2:**
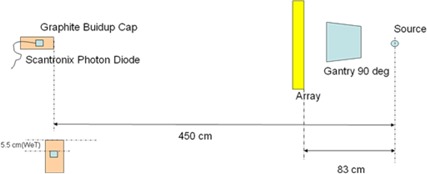
Schematics for the measurement of the narrow beam attenuation effect of a Calypso array panel. The lower left diagram displays the side view of the diode and the miniphantom.

The effects of the Calypso array panel on the photon beams were measured for a Varian Clinac iX machine. The linac was set up to deliver 6 MV and 15 MV photon beams. The beam attenuation effects were first assessed using narrow‐beam geometry. The collimator was set at 1 cm×1 cm field size. To avoid scattering interference, the detector was placed at 450 cm source detector distance (SDD), as shown in Fig. 2. A Scanditronix photon diode detector (IBA Dosimetry, Bartlett, TN) inserted into a graphite cylindrical miniphantom was used to measure the dose. The graphite phantom was 2.5 cm in diameter and had a buildup layer of 5.5 cm water‐equivalent thickness. The gantry was rotated to 90°. The Calypso array intercepted the beam line with a typical 83 cm SourceSurface Distance (SSD). The cross mark on the array surface was lined up to the linac crosshair. The longer axis of the array is normal to the gantry rotation plane. The measurement was performed with the gantry and the diode fixed in space while rotating the Calypso array panel around its longer axis from 0° to 90° at 10° intervals. Therefore, the photon beams were projected on and exited the panel at various angles. At initial position, the array surface was perpendicular to the incident beam and at final position the array surface was parallel to the incident beam. The initial position represents the gantry angle at 0° during the treatment. One should keep in mind that during patient treatment, the beam never penetrates through the entire width of the array panel as the array is always placed at a small distance above the patient. The open beam property was also measured when the array was not in the beam. The ratio of the dose measured with and without the array is the attenuation of the array to the narrow beam.

The in‐air broad beam attenuation effect of the array panel was measured with a 10 cm×10 cm field for both 6 MV and 15 MV photon beams. A 0.6 cc cylindrical Farmers chamber PTW30006 (PTW, Freiburg, Germany) with a 3 cm Lucite buildup cap was positioned at the beam isocenter. The array was placed with a gap of 2 cm, 5 cm, and 10 cm from the chamber. These gap values were selected based on observations at clinical patient setups. This measurement setup not only measures the direct attenuation to the beam from the array, it also reflects the in‐air scattering effect of the array to the beam. The gantry was rotated around the isocenter from 0° towards 90° until the 10 cm×10 cm field completely projected outside of the array. The ion chamber reading was recorded at every 10° gantry angle interval. An open beam reading was also recorded without the array in place. The ratio of the two yielded the in‐air broad beam attenuation of the array at various beam incident angles.

The beam spoiling effect of the array panel was also studied by measuring the change in percentage depth dose (PDD). An embedded Markus parallel plate chamber (PTW, Freiburg, Germany) was used to measure the PDD in a plastic water phantom (CIRS, Norfolk, VA) at 100 cm SSD. The bottom of the array panel was kept at a fixed distance of 2 cm, 5 cm, and 10 cm from the surface of the phantom. The measurements were performed with and without the array in place for both 6 MV and 15 MV photon beams.

The dose effects caused by the Calypso array panel in four typical patient treatment plans were also evaluated to determine the clinical implications. IMRT treatment plans for one prostate case and one pelvis case and two prostate VMAT cases were used for this study. The plans were all planned in Eclipse treatment planning system (Varian Medical Systems). One IMRT plan has seven beams and the other IMRT plan has nine beams distributed around the patients. One of the VMAT plans used two 286° arcs and the other used two 210° arcs. The summary of the plans is listed in Table 1. The evaluation was performed with an ArcCHECK QA device (Sun Nuclear, Melbourne, FL). The ArcCHECK QA device has a 3D diode array that is embedded in a cylindrical water‐equivalent phantom with a 3.3 cm water‐equivalent depth. These diodes have the capability to measure the photon beam *en face* as the gantry rotates during the IMRT or VMAT beam delivery. The Calypso array panel was placed at a typical distance 2 cm above the QA phantom. With this setup, three of the beams in the two IMRT plans irradiated the phantom through the Calypso array. For the VMAT plans, the array intercepts the beam central axis between beam angles 312° and 48° which are 34% and 46% of the arcs. The differences in the diode dose measured with and without the array in place were recorded.

**Table 1:** Summary of the patient plans for measuring the dose effects from the Calypso array.

*Patient*	*Plan*	*Energy*	*Beam Arrangements with Gantry Angles*
1	Prostate IMRT	6 MV	7 beams with beam angles at 156°, 104°, 52°, 0°, 308°, 256°, 200°
2	Pelvis IMRT	15 MV	9 beams with beam angles at 160°, 120°, 80°, 40°, 0°, 320°, 280°, 240°, 200°
3	Prostate VMAT	6 MV	two 286° arcs with end points at 144° and 218°
4	Prostate VMAT	6 MV	two 210° arcs with end points at 105° and 255°

## III. RESULTS & DISCUSSION

### A. Primary beam attenuation

**Figure 3:**
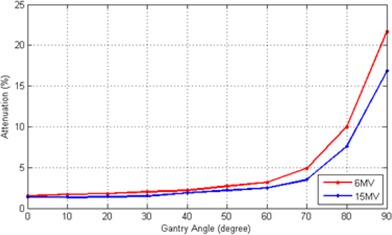
Narrow beam attenuation for 6 MV and 15 MV photon beams as a function of gantry angle. (Gantry of 0° represents normal incident beam angle to the array surface.)

The narrow beam attenuation due to the Calypso array is plotted in Fig. 3. The measurements were repeated three times at each rotation angle for each beam energy. The readings were consistent with standard deviation <1%. From Fig. 3, the attenuation to the narrow photon beam is quite small and about 2%–3% for both energies at angles less than 40° and starts to increase slowly at angles around 50°–65°. It has a sharp rise in the value at angles larger than 70° as the beam penetrated through a large portion of the array.

### B. Broad beam attenuation

**Figure 4:**
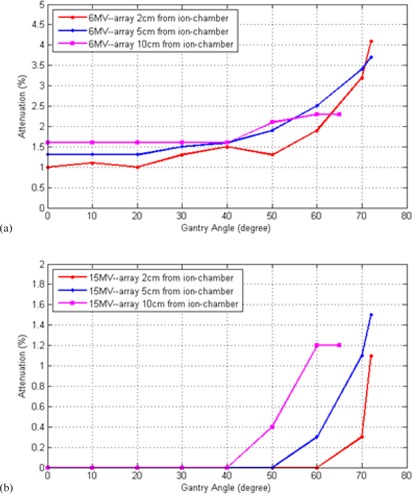
Broad beam attenuation for gap distances of 2, 5, and 10 cm between the array and the ion chamber for (a) 6 MV and (b) 15 MV photon beams as a function of beam angle. (Gantry of 0° represents normal incident beam angle to the array surface.)

Figures 4(a) and 4(b) display the broad beam angular attenuation to 6 MV and 15 MV photon beams at various array‐chamber distances. For 6 MV beam, the attenuation effect of the beam is between 1%–2% at gantry angles of 0°–40° and increases sharply up to 4.2% with gantry angles larger than 50°. In this figure, there were some fluctuations in the attenuation measurements when the array was 2 cm away from the ion chamber. This could have resulted from the inhomogeneous internal structure of the array panel. For 15 MV beam, the attenuation effect is <1% for gantry angles of 0°–40° and increases up to ∼1.5% at larger gantry angles. The range of the angles where the array intercepted and attenuated the beam decreases as the distance of the panel to the isocenter increases. The measured array attenuation for broad beam was similar to that observed by Santanam et al.^(21)^ at normal incident angle and 60° oblique angle.

### C. Beam spoiling effect

**Figure 5:**
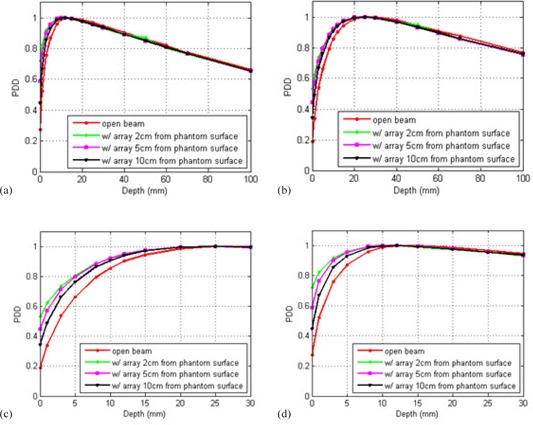
Differences in percent depth dose (PDD) for open beam and beam with array placed at 2, 5, and 10 cm from the phantom surface for (a) 6 MV and (b) 15 MV photon beam; (c) and (d) are the close‐ups of the PDD buildup regions for 6 MV and 15 MV photon beam, respectively.

The beam spoiling effects from the Calypso array are shown in Fig. 5. It is observed that the maximum depth‐dose points $d_{\text{max}}$ maintain at 1.5 cm for 6 MV and 2.5 cm for 15 MV with the array panel in place. The PDD values within the buildup region in the phantom were higher with the array than those without the array. The shorter the array‐phantom distance, the larger the PDD differences in the buildup region. In the buildup region, the differences are the largest at phantom surface and gradually decrease as depth increases. This effect is due to the scattering effect of the array. At depths beyond the maximum depth‐dose point, the differences in PDD caused by the array are smaller than 2% for both beam energies.

### D. Effects on IMRT and VMAT plan delivery

**Figure 6:**
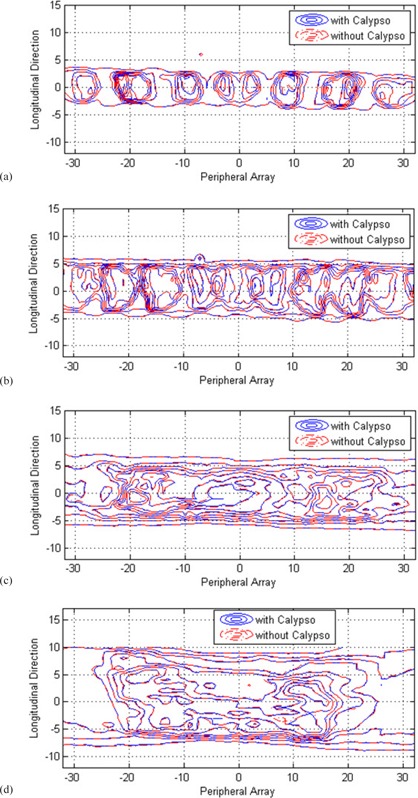
Isodose curves from patients 1 (a) and 2 (b), IMRT plan deliveries to the ArcCHECK phantom. Isodose curves from patient 3 (c) and 4 (d), VMAT plan deliveries to the ArcCHECK phantom.

**Figure 7:**
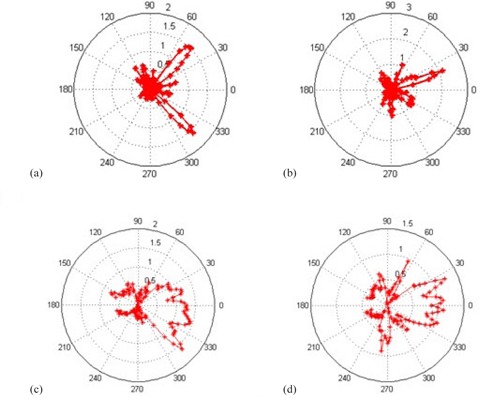
ArcCHECK phantom diode dose differences in polar plots from patient 1 (a) and 2 (b), IMRT plan deliveries. ArcCHECK phantom diode dose differences in polar plots from patient 3 (c) and 4 (d), VMAT plan deliveries.

Dose received from the diode matrix embedded in the ArcCHECK phantom was recorded for two patient IMRT plans and two patient VMAT plans. As plotted in Figs. 6(a) through 6(d), the phantom dose distributions with and without the array panel in place are close to identical. Figures 7(a) to 7(d) plot the measured dose differences caused by the array in polar plots. Three delivered plans (Figs. 7(a), 7(c), c(7)) show less than 1.6% differences in the diode readings, and the fourth plan (Fig. 7(b)) shows differences less than 2.5%. The differences were observed at the range of the beam angles where the array panel intercepted the beams. The proximal diodes showed larger differences and the distal diodes showed smaller differences as the beam penetrated through the array and the phantom.

The dose effects from the Calypso array were measured by the diode matrix embedded at 3.3 cm water‐equivalent depth in an ArcCHECK cyclindrical phantom. Recently, software packages 3DVH (Sun Nuclear Corp.) and Compass (IBA Dosimetry) were introduced to derive the patient 3D dose from the phantom dose measurements.^25^, ^26^ Nelms et al.^(27)^ published an algorithm to derive the patient high grid dose distribution from ArcCHECK phantom measurements. Future work can be performed using these methods to detail the impact of the Calypso array on patient target and OAR dose distributions from the phantom measurements.

The effect of the Calypso array on patient dose distribution can also be estimated in treatment planning system similar to the methods proposed for the evaluation of the dose effects from a treatment couch.^22^, ^23^ The CT scans of the Calypso array can be merged with the patient CT data either in the treatment planning system or other scripting languages. The dose calculation engine in the treatment planning system can be invoked to calculate the patient dose distributions that account for the effect of the Calypso array.

## IV. CONCLUSIONS

We presented here the effects of the Calypso electromagnetic array on clinical beams used on a Varian Clinac iX machine. For most clinical broad‐beam and treatment‐beam angles, the dose attenuations were measured to be within 1%–2%. The beam spoiling effect could be appreciable in the beam buildup region, but is small at deeper depths. In clinical applications for pelvis, abdomen, and thorax treatments where the tumor is at depth beyond $d_{\text{max}}$, the array effect is small and is further reduced from clinical treatment plans with multiple beams distributed around patient. Less than 2.5% dose difference on QA phantom measurements was demonstrated for patient IMRT and VMAT plans delivered with Calypso array. The results indicate that the dose difference due to the placement of Calypso array is clinically insignificant.
